# Optimizing genomic selection for blight resistance in American chestnut backcross populations: A trade‐off with American chestnut ancestry implies resistance is polygenic

**DOI:** 10.1111/eva.12886

**Published:** 2019-12-29

**Authors:** Jared W. Westbrook, Qian Zhang, Mihir K. Mandal, Eric V. Jenkins, Laura E. Barth, Jerry W. Jenkins, Jane Grimwood, Jeremy Schmutz, Jason A. Holliday

**Affiliations:** ^1^ The American Chestnut Foundation Asheville NC USA; ^2^ Department of Forest Resources and Environmental Conservation Virginia Tech Blacksburg VA USA; ^3^ Department of Biology Claflin University Orangeburg SC USA; ^4^ HudsonAlpha Institute for Biotechnology Huntsville AL USA

**Keywords:** backcross breeding, *Castanea dentata*, *Cryphonectria parasitica*, genomic selection, single‐step HBLUP

## Abstract

American chestnut was once a foundation species of eastern North American forests, but was rendered functionally extinct in the early 20th century by an exotic fungal blight (*Cryphonectria parasitica*). Over the past 30 years, the American Chestnut Foundation (TACF) has pursued backcross breeding to generate hybrids that combine the timber‐type form of American chestnut with the blight resistance of Chinese chestnut based on a hypothesis of major gene resistance. To accelerate selection within two backcross populations that descended from two Chinese chestnuts, we developed genomic prediction models for five presence/absence blight phenotypes of 1,230 BC_3_F_2_ selection candidates and average canker severity of their BC_3_F_3_ progeny. We also genotyped pure Chinese and American chestnut reference panels to estimate the proportion of BC_3_F_2_ genomes inherited from parent species. We found that genomic prediction from a method that assumes an infinitesimal model of inheritance (HBLUP) has similar accuracy to a method that tends to perform well for traits controlled by major genes (Bayes C). Furthermore, the proportion of BC_3_F_2_ trees' genomes inherited from American chestnut was negatively correlated with the blight resistance of these trees and their progeny. On average, selected BC_3_F_2_ trees inherited 83% of their genome from American chestnut and have blight resistance that is intermediate between F_1_ hybrids and American chestnut. Results suggest polygenic inheritance of blight resistance. The blight resistance of restoration populations will be enhanced through recurrent selection, by advancing additional sources of resistance through fewer backcross generations, and by potentially by breeding with transgenic blight‐tolerant trees.

## 1 INTRODUCTION

### 1.1 Historical background

Efforts to restore the American chestnut (*Castanea dentata*) have been ongoing for nearly 100 years. The chestnut blight fungus (*Cryphonectria parasitica*), first introduced into North America from Asia in the early 1900s, killed approximately 4.2 billion *C. dentata* stems from northern Mississippi to coastal Maine by the 1950s (Gravatt, 1949; Hepting, 1974; Little, 1977; Newhouse, 1990). The extirpation of *C. dentata* reduced wildlife carrying capacity and altered nutrient cycling in forests throughout its native range (Dalgleish & Swihart, 2012; Ellison et al., 2005). Today, an estimated 431 million American chestnut stems survive as seedlings and collar sprouts, but their stems rarely flower and almost never produce viable seed before being re‐infected with the blight (Dalgleish, Nelson, Scrivani, & Jacobs, 2016). Publicly funded breeding programs, initiated in the 1920s by the U.S. Department of Agriculture and the Brooklyn Botanical Garden, hybridized *C. dentata* with Asian *Castanea* species that are tolerant of chestnut blight (Anagnostakis, 2012; Burnham, Rutter, & French, 1986). However, these F_1_ hybrids were not sufficiently competitive in the mixed hardwood forests typical of the historical *C. dentata* range (Schlarbaum, Hebard, Spaine, & Kamalay, 1998), and these early chestnut breeding programs were largely discontinued by the 1960s (Jaynes, 1978).

In 1983, the American Chestnut Foundation (TACF) was founded and backcross breeding was proposed to generate hybrids that combined the blight resistance of Chinese chestnut (*Castanea mollissima*) with the timber‐type form of American chestnut (Burnham, 1981, 1988; Burnham et al., 1986). Backcrossing *C. mollissima* × *C. dentata* hybrids to *C. dentata* over three generations was expected to generate BC_3_F_1_ hybrids that inherited an average of 15/16ths (93.75%) of their genome from *C. dentata*. The BC_3_F_1_ trees were intercrossed to generate BC_3_F_2_ populations from which a subset of trees was predicted to be homozygous for blight resistance alleles from *C. mollissima*. Large quantities of blight‐tolerant BC_3_F_3_ seed for restoration would then be generated through open pollination among the selected homozygous blight‐tolerant BC_3_F_2_ trees.

The backcross method was initially implemented based on two hypotheses. First, alleles for blight resistance segregate at a few loci with incomplete dominance. Second, trees that are heterozygous for blight resistance at all loci can be reliably selected in each backcross generation. Incomplete dominance of blight resistance was surmised from the observation that F_1_ hybrids develop blight cankers that are intermediate in size and severity between *C. mollissima* and *C. dentata* (Graves, 1950). Burnham et al. (1986) hypothesized that blight resistance segregates at two loci based on observations of Clapper (1952) that F_1_ hybrids backcrossed to *C. mollissima* segregate at a ratio of three small cankered trees (blight‐tolerant) to one large cankered tree (susceptible). Later, Kubisiak et al. (1997, 2013) found that three QTLs on three linkage groups (B, F, and G) explained 40% of the variation in canker severity in a full‐sib (*C. dentata* × *C. mollissima*) × (*C. dentata* × *C. mollissima*) F_2_ family.

**Figure 1:**
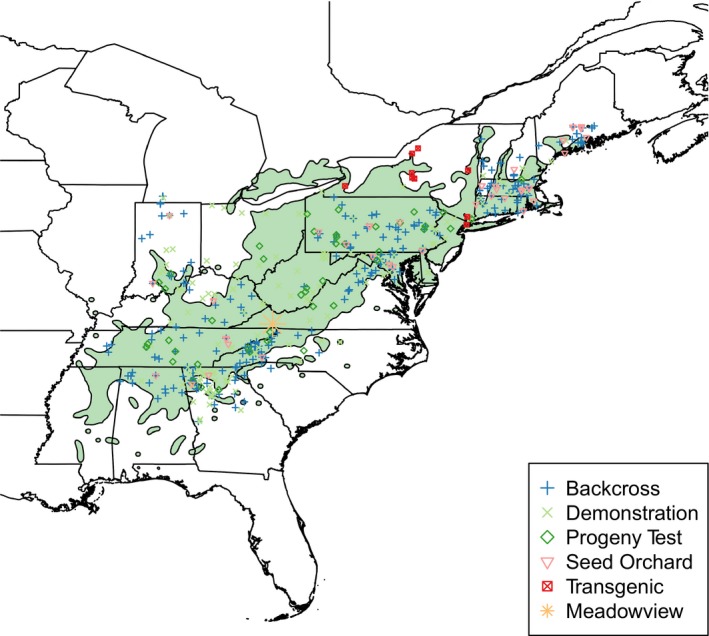
Map of the American Chestnut Foundation orchard locations across the native range of *Castanea dentata*

The American Chestnut Foundation began backcross breeding in 1989 by pollinating two (*C. dentata* × *C. mollissima*) × *C. dentata* BC_1_ hybrids (the “Clapper” and “Graves” trees) with *C. dentata* pollen from multiple trees in southwest Virginia (Hebard, 2006; Steiner et al., 2017). These BC_1_ trees were chosen as sources of blight resistance to reduce the number of additional generations of breeding and selection required to reach the BC_3_F_3_ generation. The “Clapper” and “Graves” trees have different *C. mollissima* grandparents (Clapper, 1963; Hebard, 2006) and were bred as distinct sources of resistance based on the possibility that blight resistance would segregate at different loci among the progeny of these trees. Phenotypic selection was performed in the BC_2_F_1_ and BC_3_F_1_ generations at TACF's Research Farms in Meadowview, Virginia, by artificially inoculating stems with *C. parasitica* and selecting trees with subjective canker severity ratings that were indistinguishable from F_1_ hybrids (Steiner et al., 2017). Additional selection was made for leaf and twig characteristics that resembled those of *C. dentata* (Diskin, Steiner, & Hebard, 2006; Hebard, 1994). Citizen scientists affiliated with TACF have subsequently pollinated wild‐type trees ranging from Alabama to Maine with pollen from selected BC_2_F_1_ and BC_3_F_1_ trees from the Meadowview breeding program to increase the genetic diversity and adaptive capacity of backcross populations (Westbrook, 2018; Figure 1).

### 1.2 Current state of the Meadowview breeding program

The Meadowview backcross breeding program is now reaching the final stages of selection for blight resistance. Large segregating BC_3_F_2_ populations have been generated by open pollination among selected BC_3_F_1_ descendants of the “Clapper” and “Graves” trees. Between 2002 and 2018, approximately 36,000 BC_3_F_2_ progeny of 83 “Clapper” BC_3_F_1_ selections and 28,000 BC_3_F_2_ progeny of 68 “Graves” BC_3_F_1_ selections were planted in two seed orchards. Each seed orchard contains nine blocks, and each block contains family plots of 150 BC_3_F_2_ half‐sib progeny from each *C. dentata* backcross line. Backcross lines correspond to the *C. dentata* grandparent of the BC_3_F_1_ selections, and there are 25 backcross lines in the “Graves” seed orchard and 29 backcross lines in the “Clapper” orchard (Steiner et al., 2017). Assuming that blight resistance segregates at three unlinked loci (Kubisiak et al., 1997), that all BC_3_F_1_ selections were heterozygous for *C. mollissima* alleles at these loci, and that 80% of BC_3_F_2_ seeds planted would survive to inoculation, Hebard (1994, 2002) surmised that there is a 99% probability of generating nine homozygous blight‐tolerant BC_3_F_2_ trees from each backcross line.

Between 60% and 80% of BC_3_F_2_ trees were culled on the basis of significant canker expansion 6 months after inoculation. Additional culling was performed based on late‐developing blight phenotypes that tend to be expressed in trees 5 years and older such as the survival of the main inoculated stem and the severity of additional cankers that developed as a result of natural infection by *C. parasitica* (Hebard, 2006). As of 2018, approximately 3,300 “Clapper” and 4,300 “Graves” BC_3_F_2_ trees remain. To accurately estimate the genetic resistance of the remaining BC_3_F_2_ selection candidates, TACF has planted randomized field trials of their open‐pollinated BC_3_F_3_ progeny. After inoculating these trials with *C. parasitica*, average canker severity of the most blight‐tolerant BC_3_F_3_ families was intermediate between Chinese chestnut and American chestnut. This finding led Steiner et al. (2017) to hypothesize that blight resistance segregates at more loci than previously assumed and that phenotypic selection has not been sufficiently accurate to select for the complete set of resistance alleles from *C. mollissima* founders in all backcross lines.

### 1.3 Rationale for genomic selection

The final objective for the Meadowview seed orchards is to cull all but 1% of the most blight‐resistant BC_3_F_2_ parents (i.e., select approximately 600 trees). The aim of selection is to enhance the average blight resistance of the BC_3_F_3_ progeny derived from intercrossing the BC_3_F_2_ selections, while also representing genetic diversity from most *C. dentata* parents among the selections. Progeny testing all 7,600 remaining BC_3_F_2_ selection candidates is not feasible as it would require planting hundreds of thousands of progeny and waiting many years for all BC_3_F_2_ selection candidates to flower.

In this study, we developed genomic prediction models for blight resistance to accelerate final selections. Model development entailed genotyping a training population of BC_3_F_2_ mothers whose progeny have inoculated with *C. parasitica*. The blight resistance of the unknown fathers of the BC_3_F_3_ progeny may have biased the progeny canker severity breeding value estimates, especially for small BC_3_F_3_ families. To control for this potential bias, we also used genomic relationships among BC_3_F_2_ trees to estimate breeding values for five late‐developing blight phenotypes for individual selection candidates. We summed the breeding values for the late‐developing phenotypes of the BC_3_F_2_ trees with breeding values for average canker severity of their BC_3_F_3_ progeny to estimate a Blight Selection Index for each selection candidate. The selection index enabled simultaneous ranking of a large number of BC_3_F_2_ selection candidates including trees <5 years old that have not flowered and were too young to reliably express blight resistance phenotypes.

### 1.4 Study objectives

Our first aim was to optimize an analytical pipeline for genomic selection for blight resistance in American chestnut backcross populations. Toward this end, we generated a draft reference genome for *C. dentata* and performed genotyping by sequencing on 1,230 BC_3_F_2_ selection candidates from the Meadowview breeding program. We optimized the single‐step (HBLUP) method, in which breeding values were predicted from a blend of pedigree and genomic relationships with trees in the training population (Aguilar et al., 2009; Legarra, Aguilar, & Misztal, 2009; Misztal, Legarra, & Aguilar, 2009).

Our second aim was to investigate the genetic architecture of blight resistance by estimating the correlation between the proportion of BC_3_F_2_ trees' genomes inherited from *C. dentata* and breeding values for blight resistance. Hypothetically, a major gene architecture would be implied if a subset of BC_3_F_2_ trees demonstrated high blight resistance and inherited a high percentage (>90%) of their genome from *C. dentata*. By contrast, a strong negative correlation between blight resistance and genome inheritance from *C. dentata* would suggest polygenic control. We also compared the predictive ability of HBLUP to Bayes C regression. Bayes C, which includes only the largest effect markers in the prediction model, has been found to have greater predictive ability than HBLUP for traits that are controlled by few major effect loci. In contrast, HBLUP and Bayes C have similar predictive ability for polygenic traits (Chen, Li, Sargolzaei, Schenkel, 2014; Resende et al., 2012; Yoshida et al., 2018).

## 2 MATERIALS AND METHODS

### 2.1 Phenotyping

#### 2.1.1 Phenotyping BC_3_F_3_ progeny

Between 2011 and 2016, 7,173 BC_3_F_3_ progeny from 346 “Clapper” and 198 “Graves” open‐pollinated BC_3_F_2_ mothers were evaluated for blight resistance. Between 27 and 33 BC_3_F_3_ progeny from each BC_3_F_2_ mother were planted at TACF's Meadowview Research Farms in a completely randomized design (2011–2013 tests) or an alpha‐lattice incomplete block design (2014–2016 tests; Patterson & Williams, 1976). In their third growing season, the main stems of BC_3_F_3_ trees were inoculated with the SG2,3 (weakly pathogenic) and Ep155 (highly pathogenic) strains of *C. parasitica* at two stem heights approximately 25 cm apart using the cork borer agar disk method (TACF, 2016). The SG2,3 and Ep155 strains were originally isolated from American chestnut trees in Virginia and Maryland, respectively (M. Double, personal communication). The BC_3_F_3_ family rankings for average canker severity using these two strains were strongly genetically correlated (*r*_genetic_ > 0.95), suggesting generalized rather than strain‐specific mechanisms of host blight resistance (Steiner et al., 2017; Westbrook & Jarrett, 2018).

Canker lengths and subjective ratings were phenotyped 5–6 months after inoculation. Cankers were rated as 1 = minimal expansion beyond initial lesion, 2 = some expansion, but canker partially contained by callus formation, or 3 = canker large, sunken, and sporulating (Figure S1). The trait “canker severity” was calculated separately for each strain of *C. parasitica* (SG2,3 & Ep155) by scaling the variation in canker lengths and canker ratings to mean 0 and standard deviation 1, and summing the standardized rating and length. The canker severities for each strain of *C. parasitica* were then summed to obtain a single canker severity value for each tree. Canker severity phenotypes were obtained for 48% of the BC_3_F_3_ seeds that were planted, and 2–40 BC_3_F_3_ progeny (median = 13) were phenotyped per BC_3_F_2_ mother. Canker severity phenotypes of BC_3_F_3_ trees were continuously distributed, and there was no difference in the average canker severity in the Clapper and Graves BC_3_F_3_ populations (Figure S2).

#### 2.1.2 Phenotyping BC_3_F_2_ selection candidates

Trees remaining in Meadowview seed orchards that were between 5 and 16 years old were phenotyped for five binary traits hypothesized to be indicative of blight resistance or susceptibility. All trees were phenotyped for main stem survival. Trees with a living main stem were then phenotyped for four additional traits on the main stem, namely the presence or absence of any canker longer than 15 cm; the presence or absence of exposed wood; the presence or absence of sporulation of *C. parasitica* conidia from cankers; and the presence or absence of sunken cankers. In total, 1,134 “Clapper” and 1,042 “Graves” BC_3_F_2_ selection candidates were phenotyped for these traits.

### 2.2 Genetic marker development and genotyping

#### 2.2.1 Generation of a draft reference genome for *Castanea dentata*

We generated a draft reference genome sequence for the immediate purpose of detecting SNP variants in backcross populations. We sequenced the “Ellis1” clone of *C. dentata* by whole‐genome shotgun sequencing using the PacBio Sequel sequencing platform at the HudsonAlpha Institute in Huntsville, Alabama. A total of 16 cells using chemistry 2.1 were sequenced with a p‐read yield of 88.69 Gb (8,327,003 reads), for a total coverage of 98.54× (median read size 7,745 bp). The reads were assembled using MECAT (Xiao et al., 2017) and subsequently polished using ARROW (Chin et al., 2013). This produced 2,959 contigs with an N_50_ of 4.4 Mb and a total genome size of 967.1 Mb. Contigs were then collapsed to remove redundant alternative haplotype sequence and screened against bacterial proteins, organelle sequences, and the GenBank nonredundant database to detect and remove contaminants. Version 0.5 of the *C. dentata* genome contains 793.5 Mb of sequence, consisting of 950 contigs with a contig N_50_ of 8.1 Mb.

#### 2.2.2 Library preparation for genotyping by sequencing

Newly expanded leaves were collected from BC_3_F_2_ selection candidates in June 2017. The leaf tissue was ground in liquid nitrogen, and genomic DNA was extracted using a Qiagen DNeasy Plant Mini Kit. The quality and quantity of DNA were checked on a NanoDrop spectrophotometer (ND‐100), and 200 ng of DNA from each tree was digested with 1 µl of ApeKI. Illumina‐compatible adapters were ligated with 1.6 µl of T4 DNA ligase. Each of the P1 adapters had a variable‐length (4–8 bp) index downstream of the sequencing primer such that it was read immediately preceding the restriction site. The P2 adapter was common across all samples. Following adapter ligation, 18 cycles of PCR were performed to confirm ligation and the fragment size range. The DNA samples were randomly assigned to pools of 50 per lane for trees whose progeny had previously been inoculated with *C. parasitica* or 96 per lane for trees whose progeny had not been inoculated. Pools were purified with the New England Biolabs Monarch PCR and DNA Clean‐Up Kit before and after PCR amplification. Fragments in the range of 250–550 bp were selected on a BluePippin™ instrument (Sage Science), and the resulting libraries were visualized on a Bioanalyzer (Agilent 2100 Bioanalyzer). Libraries were then sequenced on an Illumina HiSeq 4000 instrument in 2× 150 bp paired‐end mode at the Duke University Center for Genomic and Computational Biology.

#### 2.2.3 Bioinformatics for SNP calling

Raw reads were filtered for quality, filtered for adapter contamination, and demultiplexed using STACKS software (Catchen, Hohenlohe, Bassham, Amores, & Cresko, 2013). Filtered reads were then aligned to v. 0.5 of the *C. dentata* reference genome using the Burrows‐Wheeler Aligner (BWA) *mem* algorithm and subsequently converted to BAM format, sorted, and indexed with SAMtools (Li & Durbin, 2010; Li et al., 2009). GVCF files for each sample were generated using the GATK HaplotypeCaller algorithm (McKenna et al., 2010; Poplin et al., 2017), and these GVCFs were then merged using the GenotypeGVCFs function to create a candidate polymorphism set. Variants were flagged and removed as low quality if they had the following characteristics: low map quality (MQ < 40); high strand bias (FS > 40); differential map quality between reads supporting the reference and alternative alleles (MQRankSum < −12.5); bias between the reference and alternate alleles in the position of alleles within the reads (ReadPosRankSum < −8.0); and low depth of coverage (DP < 5). The resulting VCF file was filtered to retain only biallelic SNPs with <10% missing data and minor allele frequencies >0.01, leaving 71,507 SNPs. Missing SNP genotypes were imputed with Beagle v 4.1 (Browning & Browning, 2016). A total of 1,230 (865 “Clapper” and 365 “Graves”) BC_3_F_2_ individuals were genotyped.

### 2.3 Genomic prediction and validation

#### 2.3.1 Single‐step prediction of progeny canker severity

**Figure 2:**
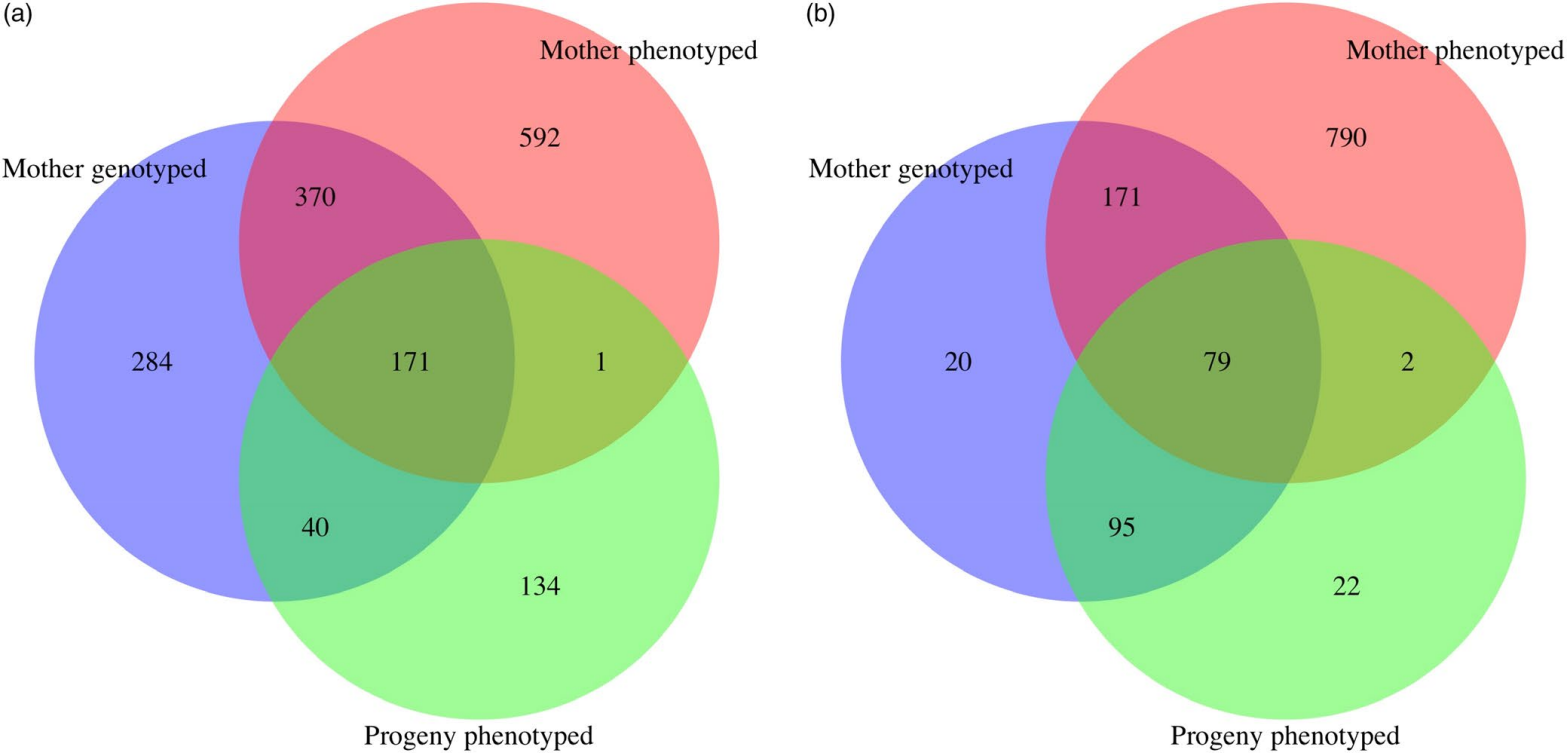
Numbers of BC_3_F_2_ selection candidates from the “Clapper” and “Graves” populations that were phenotyped for five traits indicative of blight resistance (Mother phenotyped), whose BC_3_F_3_ progeny were phenotyped for canker severity (Progeny phenotyped), and/or were genotyped for genomic selection (Mother genotyped)

Breeding values for average progeny canker severity were obtained for all open‐pollinated BC_3_F_2_ mothers that were genotyped and/or whose BC_3_F_3_ progeny were phenotyped using the single‐step HBLUP method (Aguilar et al., 2009; Legarra et al., 2009; Misztal et al., 2009). This method blends the pedigree and genomic relationship matrix into a single matrix **H** so that phenotypic and genotypic data for both genotyped and nongenotyped individuals can be used to estimate breeding values. Breeding values were estimated from blended pedigree and genomic relationships and progeny canker severity phenotypes for 211 “Clapper” and 154 “Graves” BC_3_F_2_ mothers; from pedigree relationships and progeny phenotypes for 135 “Clapper” and 44 “Graves” BC_3_F_2_ mothers that died prior to genotyping; and from pedigree and genomic relationships alone for 654 “Clapper” and 211 “Graves” BC_3_F_2_ mothers whose progeny had not yet been phenotyped (Figure 2). Single‐step prediction of average progeny canker severity was first performed separately for “Clapper” and “Graves” BC_3_F_2_ populations based on the assumption that these populations are unrelated. Then, the data from both populations were combined into a single analysis to determine whether realized genomic relatedness between populations enhanced predictive ability.

Martini et al. (2018) found that the parameters *τ* and ω that scale the inverse of genomic and pedigree relationships respectively in **H^−1^** influence predictive ability and bias in prediction of breeding values. We therefore performed single‐step prediction with H‐matrices parameterized with nine pairwise combinations of *τ* and *ω* involving *τ* = 1, 2, or 3 and *ω* = 1, 0, or −1 and a tenth combination in which *τ* = *ω* = 0, which is equivalent to the pedigree relationship matrix. We sought the combination of *τ* and *ω* that maximized predictive ability while minimizing prediction bias. The inverse of the parameterized H‐matrix (hereafter referred to as $H_{\tau ,\omega }^{-1}$) was calculated following Martini et al., (2018):

(1) $$H_{\tau ,\omega }^{-1}=A^{-1}+\left[\begin{array}{cc} 0 & 0\\ 0 & \left(\tau G^{-1}+\omega A_{22}^{-1}\right) \end{array}\right]$$

where **A^−1^** is the inverse of the pedigree relationship matrix, **G^−1^** is the inverse genomic relationship matrix, and $A_{22}^{-1}$ is the inverse pedigree relationship matrix among genotyped individuals. Genomic relationships in **G** were estimated following VanRaden (2008):

(2) $$G={\mathrm{ZZ}}^{\prime }/2\sum_{j=1}^{j}p_{j}\left(1-p_{j}\right)$$

where **Z** is the centered genotypic matrix, and *p_j_* are reference allele frequencies for locus 1 through *j*.

Breeding values of average progeny canker severity for BC_3_F_2_ mothers were estimated with different parameterizations of $H_{\tau ,\omega }^{-1}$ with the following model in ASReml‐R v. 4.1 (Butler, Cullis, Gilmour, & Gogel, 2018):

(3) $$y_{\mathrm{ijkl}}=\mu +t_{i}+r_{j\left(i\right)}+b_{k\left(\mathrm{ji}\right)}+g_{l}+\varepsilon_{\mathrm{ijkl}}$$

where *y* is a vector of canker severity phenotypes for individual BC_3_F_3_ progeny, and μ is the trait mean. The vector *t_i_* is composed of the random effects of inoculation years (2011–2016) that were assumed to be independently and normally distributed ($t_{i}∼N\left[0,I\sigma_{t}^{2}\right]$, **I** is an identity matrix); *r_j_*_(_ *_i_*_)_ are random effects of complete blocks within the years (2014–2016 trials only) ($r_{j\left(i\right)}∼N\left[0,I\sigma_{r}^{2}\right]$); *b_k_*_(_ *_ij_*_)_ are the random effects of incomplete block within the complete block and year (2014–2016 trials only) ($b_{k\left(\mathrm{ij}\right)}∼N[0,I\sigma_{b}^{2}$]); *g_l_* are the random additive genetic effects (i.e., the breeding values) of BC_3_F_2_ mothers ($g_{l}∼N\left[0,H_{\tau ,\omega }\sigma_{g}^{2}\right]$); and ε_ijkl_ are the residuals ($\varepsilon_{\mathrm{ijkl}}∼N[0,I\sigma_{e}^{2}$]). Residuals were approximately normally distributed, and no data transformation was performed. The heritability of family mean canker severity ($h_{\text{family}}^{2}$) among BC_3_F_3_ progeny of open‐pollinated BC_3_F_2_ selection candidates was estimated as follows:

(4) $$h_{\text{family}}^{2}=\frac{\sigma_{g}^{2}}{\left(\sigma_{g}^{2}+\sigma_{e}^{2}/n\right)}$$

where *n* = 13.5 is the mean number BC_3_F_3_ progeny evaluated per BC_3_F_2_ mother tree (Isik, Holland, & Maltecca, 2017).

The accuracy of single‐step prediction of breeding values for progeny canker severity was estimated with 10‐fold cross‐validation. The cross‐validation was performed in ASReml‐R by randomly subdividing the phenotyped BC_3_F_3_ families into ten subsets and using phenotypic data from 9/10ths of the families to predict breeding values for the remaining 1/10th of the families via $H_{\tau ,\omega }^{-1}$. This procedure was repeated for each subset of families to obtain predicted breeding values for all BC_3_F_2_ mothers. Predictive ability ($r_{y\hat{y}}$) of family mean canker severity was estimated as follows:

(5) $$r_{y\hat{y}}=r_{g\hat{g}}\times \sqrt{h_{\text{family}}^{2}}$$

where $r_{g\hat{g}}$ is the Pearson correlation between predicted breeding values and breeding values estimated with phenotypes from all families (Resende et al., 2012). The entire 10‐fold cross‐validation procedure was repeated ten times for each parameterization $H_{\tau ,\omega }^{-1}$ to estimate variation in predictive ability that arises from randomly subdividing the training population into training and prediction subsets. The same random partitions were used for each combination of *τ* and *ω* to compare predictive ability with different parameterizations of $H_{\tau ,\omega }^{-1}$.

Bias in prediction of breeding values was estimated by regressing adjusted family mean canker severity (*y*‐axis) on the predicted breeding values for progeny canker severity (*x*‐axis; Martini et al., 2018; Piñeiro, Perelman, Guerschman, & Paruelo, 2008). A regression coefficient *b* < 1 indicated inflation of predicted breeding values relative to family means. Percent bias was calculated as 1 − *b* * 100. Bias was deemed acceptable if the percent bias intersected zero among cross‐validation replicates. The R script for single‐step prediction of average progeny canker severity is included in Supporting Information.

#### 2.3.2 Comparing HBLUP to Bayes C

The accuracy of the optimized HBLUP procedure was compared to that of Bayes C and prediction from pedigree relationships (ABLUP); Bayes C first estimates the parameter π, which is the proportion of SNPs with nonzero effects, and then estimates allelic substitution effects of these SNPs assuming that the effects are normally distributed (Habier, Fernando, Kizilkaya, & Garrick, 2011). Bayes C was implemented with the R package BGLR (Perez & de los Campos, 2014). Marker effects were estimated over 50,000 iterations of a Gibbs sampler after 10,000 burn‐in iterations. Residual plots were inspected to confirm that there was no autocorrelation between iterations (Perez & de los Campos, 2014).

To perform 10‐fold cross‐validation with Bayes C, allelic substitution effects were estimated on adjusted family mean canker severity for 9/10th of the training population. Adjusted family means were estimated in ASReml‐R by treating BC_3_F_2_ mothers as fixed factors and year, block, and incomplete block as random factors as in Equation 3. Breeding values ($\hat{g}$) for the remaining 1/10th of the population were predicted with the formula:

(6) $$\hat{g}=\mu +\sum_{i=1}^{N}Z\hat{m}$$

where is **Z** is the centered and imputed genotypic matrix, *N* is the number of SNPs with minor allele frequency >0.01, and $\hat{m}$ is a vector of allelic substitution effects. Family mean prediction accuracy ($r_{\hat{y}\hat{g}}$), or the Pearson correlation between predicted breeding values and adjusted family mean canker severity, was used to compare the accuracy of Bayes C, HBLUP, and ABLUP. The 10‐fold cross‐validation was repeated ten times with different random partitions of the training population to estimate variation in predictive ability due to training population composition.

#### 2.3.3 Genomic prediction of binary blight phenotypes of BC_3_F_2_ parents

Single‐step analysis of the blight phenotypes of BC_3_F_2_ selection candidates was performed (a) to estimate the heritability of these phenotypes and (b) to predict breeding values for genotyped trees aged five or less that were too young to reliably express these phenotypes. Breeding values for these traits were predicted for 324 “Clapper” and 115 “Graves” BC_3_F_2_ trees that were aged five or younger from genomic or pedigree relationships with 1,134 Clapper and 1,042 Graves BC_3_F_2_ trees that were phenotyped (Figure 2). Breeding values and heritability of presence/absence blight phenotypes of individual BC_3_F_2_ trees were estimated with the binomial mixed model:

(7) $$y_{\mathrm{ijk}}=\mu +t_{i}+b_{j}+g_{k}+\varepsilon_{\mathrm{ijk}}$$

where *y* is a binary phenotype (i.e., main stem alive/dead, presence/absence of large cankers, exposed wood, sporulation, or sunken cankers); $t_{i}∼N\left[0,I\sigma_{t}^{2}\right]$ are the random effects of years that the BC_3_F_2_ trees were planted (2002–2014); $b_{j}∼N[0,I\sigma_{b}^{2}$] are the random effects of seed orchard block (1–9); $g_{k}∼N\left[0,H_{\tau ,\omega }\sigma_{g}^{2}\right]$ are the random additive genetic effects of individual BC_3_F_2_ trees; and $\varepsilon_{\mathrm{ijk}}∼N[0,I\sigma_{e}^{2}$] are the residuals. Phenotypic classes indicative of blight resistance were coded as 1, whereas classes indicative of susceptibility were coded as 0 (e.g., main stem alive = 1 or dead = 0; large cankers absent = 1 or present = 0; exposed wood absent = 1 or present = 0; sporulation absent = 1 or present = 0; and sunken cankers absent = 1, present = 0). Heritability and prediction accuracy of breeding values were compared for two parameterizations of $H_{\tau ,\omega }^{-1}$: (a) *τ* = *ω* = 0, which is equivalent to the pedigree relationship matrix and (b) *τ* = *ω* = 1, which scales $G^{-1}$ and $A_{22}^{-1}$ equally. Given that individual trees from the BC_3_F_2_ generation were the targets of selection, we estimated the heritability of blight phenotypes of individual BC_3_F_2_ selection candidates ($h_{\text{ind}}^{2}$):

(8) $$h_{\text{ind}}^{2}=\sigma_{g}^{2}/\left(\sigma_{g}^{2}+\pi^{2}/3\right)$$

where $\pi^{2}/3$ is the variance of the standard logistic distribution (Davies, Scarpino, Pongwarin, Scott, & Matz, 2015). Breeding values for binary blight traits were estimated as probability of having a trait value of 1 given the individual trees' genotype. This probability was calculated as follows:

(9) $$p=\exp\left(\mu +g\right)/\left(1+\exp\left[\mu +g\right]\right)$$

where *μ* is the model intercept and *g* is a vector random genetic effects in units of logit scores (Gezan & Munoz, 2014).

Breeding value prediction accuracy ($r_{g\hat{g}}$) was estimated for the “Clapper” and “Graves” populations separately with 10‐fold cross‐validation. Breeding value prediction accuracy was defined as the Pearson correlation between predicted breeding values when the trees' phenotypes were left out of the model versus when all trees' phenotypes were included. The 10‐fold cross‐validation was repeated ten times for each trait with different random partitions of each population.

### 2.4 Estimation of Blight Selection Indices and hybrid indices

#### 2.4.1 Estimation of selection indices for blight resistance

A selection index called “Parent Condition Index” was created by summing the breeding values estimated for each of the five blight traits that were phenotyped in the BC_3_F_2_ population. The variance in breeding values for each trait is proportional to the trait's heritability; thus, each trait was weighted in proportion to $h_{\text{ind}}^{2}$. The breeding values for progeny canker severity were multiplied by −1 to obtain the variable “Progeny Blight Resistance.” Both Parent Condition Index and Progeny Blight Resistance were standardized to mean = 0 and standard deviation = 1 so that they would be equally weighted. The standardized variables were then summed to create the “Blight Selection Index.”

#### 2.4.2 Estimation of hybrid indices

Hybrid indices were estimated to determine whether blight resistance is correlated with proportion of the backcross trees' genomes inherited from *C. dentata*. Hybrid indices were estimated for BC_3_F_2_ trees with the R package *introgress* (Gompert & Buerkle, 2010). To generate the required parental data, genotyping by sequencing was performed as described above on 56 *C. dentata* individuals and 47 *C. mollissima* individuals. Bioinformatic processing of these data was the same as for the BC_3_F_2_ samples, and after merging data from the pure species and BC_3_F_2_ samples, 27,306 SNPs were retained. The VCF file was converted to STRUCTURE format with PLINK software (http://zzz.bwh.harvard.edu/plink/) and subsequently to *introgress* format using the prepare.data function in *introgress*. Hybrid indices and their confidence limits were then estimated using the est.h function.

## 3 RESULTS

### 3.1 Accuracy of single‐step prediction of progeny canker severity

The first step in developing genomic prediction models for blight resistance was to optimize prediction of average progeny canker severity of the open‐pollinated BC_3_F_2_ selection candidates. We compared predictive ability ($r_{y\hat{y}}$) and bias of predicted breeding values with different scaling parameters for the inverses of the genomic relationship matrix (**G^−1^**) and pedigree relationship matrix for genotyped selection candidates ($A_{22}^{-1}$) within $H_{\tau ,\omega }^{-1}$ (Equation 1). When prediction models were trained on either “Clapper” or “Graves” BC_3_F_3_ progeny separately, average predictive ability was maximized ($r_{y\hat{y}}=0.64$ for “Clapper” and $r_{y\hat{y}}=0.46$ for “Graves”) while percent bias intersected zero across cross‐validation replicates when **G^−1^** was multiplied by *τ* = 3, and $A_{22}^{-1}$ was multiplied by *ω* = 1 (Table 1). Training genomic prediction models on the “Clapper” and “Graves” BC_3_F_3_ populations combined decreased maximum predictive ability for the “Clapper” population ($r_{y\hat{y}}=0.52$) and marginally increased maximum predictive ability for the “Graves” population ($r_{y\hat{y}}=0.48$) at the parameterization of $H_{\tau ,\omega }^{-1}$ that minimized prediction bias (*τ* = 3, *ω* = 0). Genotyping revealed that some BC_3_F_2_ selection candidates were more closely related than expected from pedigree relationships (Figure S3). Average predictive ability was lower when predicting from pedigree relationships ($r_{y\hat{y}}=0.25$ for “Clapper”; $r_{y\hat{y}}=0.26$ for “Graves”) as compared with most parameterizations of $H_{\tau ,\omega }^{-1}$ (Table 1). Considering these results, we trained the genomic prediction model for the “Clapper” population separately after multiplying **G^−1^** by 3 and $A_{22}^{-1}$ by 1. We trained the genomic prediction model for “Graves” on the “Clapper” and “Graves” populations combined after multiplying **G^−1^** by 3 and $A_{22}^{-1}$ by 0.

**Table 1:** Optimizing genomic prediction of BC_3_F_3_ family mean canker severity with different parameterizations of the blended pedigree and genomic relationship matrix ($H_{\tau ,\omega }^{-1}$)

*τ*	*ω*	h^2^_family_ ± *SE*	Clapper $r_{y\hat{y}}$		Graves $r_{y\hat{y}}$		Bias %	
			Avg	Max, Min	Avg	Max, Min	Avg	Max, Min
Clapper (346 families)								
3	1	0.67 ± 0.06	0.64	0.65, 0.61	—	—	−9	2, −16
2	1	0.64 ± 0.06	0.59	0.61, 0.58	—	—	−3	8, −10
1	1	0.54 ± 0.07	0.51	0.52, 0.49	—	—	5	16, −3
3	0	0.73 ± 0.05	0.58	0.59, 0.56	—	—	−39	−28, −53
2	0	0.71 ± 0.05	0.53	0.55, 0.51	—	—	−33	−21, −48
1	0	0.68 ± 0.05	0.55	0.56, 0.51	—	—	−21	−6, −39
3	−1	0.75 ± 0.05	0.55	0.56, 0.52	—	—	−48	−32, −66
2	−1	0.74 ± 0.05	0.50	0.52, 0.47	—	—	−37	−19, −57
1	−1	0.72 ± 0.05	0.50	0.52, 0.46	—	—	−15	5, −37
0	0	0.56 ± 0.06	0.25	0.26, 0.22	—	—	49	61, 37
Graves (198 families)								
3	1	0.59 ± 0.09	—	—	0.46	0.48, 0.45	−27	6, −60
2	1	0.50 ± 0.10	—	—	0.42	0.44, 0.40	−19	12, −50
1	1	0.34 ± 0.09	—	—	0.35	0.36, 0.34	−14	16, −43
3	0	0.68 ± 0.08	—	—	0.35	0.38, 0.33	−69	−5, −133
2	0	0.65 ± 0.09	—	—	0.32	0.35, 0.29	−63	3, −126
1	0	0.58 ± 0.09	—	—	0.27	0.29, 0.24	−54	13, −113
3	−1	0.72 ± 0.08	—	—	0.33	0.36, 0.29	−92	−11, −171
2	−1	0.69 ± 0.08	—	—	0.30	0.33, 0.26	−84	−3, −158
1	−1	0.65 ± 0.09	—	—	0.27	0.29, 0.23	−69	8, −131
0	0	0.35 ± 0.10	—	—	0.24	0.26, 0.22	−13	27, −45
Clapper and Graves								
3	1	0.75 ± 0.04	0.61	0.65, 0.60	0.56	0.59, 0.52	21	26, 13
2	1	0.71 ± 0.04	0.57	0.61, 0.56	0.51	0.54, 0.47	26	31, 18
1	1	0.61 ± 0.05	0.50	0.54, 0.48	0.45	0.48, 0.41	33	38, 25
3	0	0.81 ± 0.03	0.52	0.55, 0.49	0.48	0.51, 0.43	2	12, −10
2	0	0.79 ± 0.03	0.46	0.50, 0.44	0.44	0.47, 0.39	8	20, −3
1	0	0.76 ± 0.03	0.38	0.42, 0.36	0.37	0.40, 0.33	18	33, 8
3	−1	0.83 ± 0.03	0.47	0.51, 0.45	0.46	0.49, 0.37	−1	14, −13
2	−1	0.81 ± 0.03	0.42	0.45, 0.40	0.41	0.45, 0.37	7.2	25, −4
1	−1	0.79 ± 0.03	0.35	0.37, 0.32	0.36	0.38, 0.32	21	41, 10
0	0	0.61 ± 0.04	0.18	0.20, 0.16	0.27	0.29, 0.25	66	73, 56

Note

The pedigree relationship matrix is equivalent to *τ* = *ω* = 0.

The parameters *τ* and *ω* scale the inverse of pedigree and genomic relationships among genotyped individuals, The family mean heritability ($h_{\text{family}}^{2}$), predictive ability of family means $\left(r_{y\hat{y}}\right)$, and percent prediction bias were compared when prediction models were trained on the “Clapper” and “Graves” populations separately and when both populations were combined.

### 3.2 Prediction of progeny canker severity using Bayes C

The Bayes C method, which sets a proportion of the marker effects to zero, had a similar accuracy to predict average progeny canker severity as compared with the single‐step (HBLUP) method, which incorporates all markers into the prediction (Table 2). Both and HBLUP and Bayes C methods were more accurate than prediction from the pedigree (ABLUP; Table 2). Considering these results, we used the optimized single‐step method rather than Bayes C or the pedigree to predict breeding values for progeny canker severity.

**Table 2:** Comparing the optimal blend of pedigree and genomic relationships (HBLUP), pedigree relationships (ABLUP), and the Bayes C method based on accuracy to predict observed BC_3_F_3_ family mean canker severity ($r_{\hat{y}\hat{g}}$) in 10‐fold cross‐validation repeated ten times

	Clapper						Graves					
	Proportion of markers included			$r_{\hat{y}\hat{g}}$			Proportion of markers included			$r_{\hat{y}\hat{g}}$		
	Max	Avg	Min	Max	Avg	Min	Max	Avg	Min	Max	Avg	Min
HBLUP	1	1	1	0.32	0.30	0.28	1	1	1	0.19	0.15	0.11
ABLUP	0	0	0	0.07	0.04	0.01	0	0	0	0.09	0.06	0.02
Bayes C	0.58	0.52	0.25	0.28	0.26	0.21	0.59	0.41	0.29	0.22	0.16	0.12

Note

In the Bayes C method, the proportion of markers with nonzero effects varied between cross‐validation replicates.

### 3.3 Prediction of blight phenotypes of BC_3_F_2_ selection candidates

**Figure 3:**
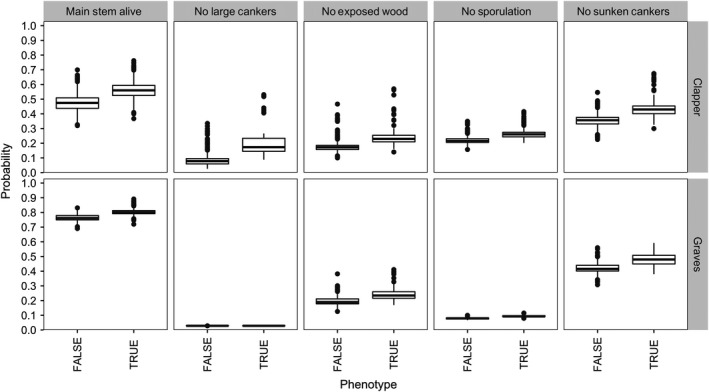
Probabilities that BC_3_F_2_ trees will have a phenotype indicative of blight resistance given trees' genotypes versus trees' observed phenotypes for “Clapper” and “Graves” BC_3_F_2_ trees

To correct for unknown paternal bias in ranking open‐pollinated BC_3_F_2_ selection candidates based on progeny canker severity, we also ranked the selection candidates based on the “Parent Condition Index” or the sum of breeding values for five late‐developing blight phenotypes of the selection candidates themselves. The blight phenotypes of individual BC_3_F_2_ selection candidates were weakly heritable, with h^2^_ind_ values varying from 0 to 0.25 depending on the trait (Table 3). On average, trees with an observed phenotype indicative of blight resistance (i.e., main stem alive) also had a greater breeding value or probability expressing a resistance phenotype (Figure 3). However, due to the low heritability of the blight phenotypes of BC_3_F_2_ selection candidates, there was substantial overlap in the distributions of breeding values between resistant versus susceptible phenotypic classes. For example, in the “Graves” population, there was no differentiation in breeding values for trees with presence or absence of large cankers and presence or absence of sporulation, indicating that these traits did not contribute to the Parent Condition Index in this population (Figure 3). Cross‐validation was performed to estimate breeding value prediction accuracy for trees younger than 5 years. Averaged across cross‐validation replicates, breeding value prediction accuracy using the H‐matrix varied from 0.88 to 0.92 for “Clapper” selection candidates and from 0.5 to 0.94 for “Graves” selection candidates. Averaged across traits, accuracy was 1.3 times greater in the “Clapper” population and 2.5 times greater in the “Graves” population when using the H‐matrix as compared with prediction from the pedigree (Table 3). Therefore, the H‐matrix was used to estimate breeding values for the Parent Condition Index.

**Table 3:** Comparing the H‐matrix and the pedigree relationship matrix estimates of individual tree heritability ($h_{\text{ind}}^{2}$ ± *SE*) and accuracy to predict breeding values ($r_{g\hat{g}}$) for five binary blight phenotypes of BC_3_F_2_ selection candidates

	N TRUE	N FALSE	Pedigree $h_{\text{ind}}^{2}$ ± *SE*	H‐matrix $h_{\text{ind}}^{2}$ ± *SE*	Pedigree accuracy $r_{g\hat{g}}$			H‐matrix accuracy $r_{g\hat{g}}$		
					Max	Avg	Min	Max	Avg	Min
Clapper										
Main stem alive	645	489	0.07 ± 0.04	0.08 ± 0.04	0.78	0.75	0.67	0.93	0.91	0.89
No large cankers	55	577	0.20 ± 0.10	0.25 ± 0.09	0.78	0.74	0.68	0.91	0.88	0.83
No sporulation	151	483	0.03 ± 0.07	0.07 ± 0.06	0.52	0.44	0.27	0.90	0.86	0.81
No exposed wood	109	519	0.11 ± 0.08	0.12 ± 0.06	0.80	0.70	0.60	0.94	0.92	0.88
No sunken cankers	245	388	0.08 ± 0.06	0.09 ± 0.05	0.76	0.72	0.67	0.92	0.90	0.89
Graves										
Main stem alive	872	170	0.08 ± 0.06	0.06 ± 0.06	0.42	0.37	0.34	0.84	0.80	0.73
No large cankers	28	843	0.05 ± 0.24	0	0.32	0.26	0.17	NA	NA	NA
No sporulation	70	802	0.04 ± 0.12	0.05 ± 0.11	0.28	0.21	0.13	0.60	0.55	0.50
No exposed wood	191	681	0.04 ± 0.05	0.07 ± 0.05	0.36	0.31	0.25	0.95	0.94	0.92
No sunken cankers	412	457	0.07 ± 0.04	0.07 ± 0.05	0.52	0.46	0.40	0.89	0.87	0.86

Note

Accuracy was assessed with 10‐fold cross‐validation repeated 10 times. Accuracy using the H‐matrix was not assessed for presence/absence of large cankers in the “Graves” population because the trait heritability was zero.

### 3.4 Estimation of hybrid indices

**Figure 4:**
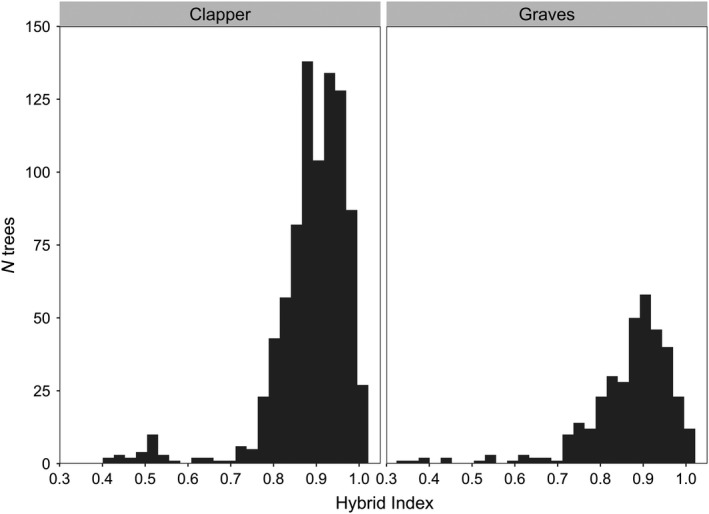
Distribution of hybrid index values for BC_3_F_2_ descendants of “Clapper” and “Graves.” Hybrid index values indicate the proportion of hybrid genomes inherited from *Castanea dentata* v. *Castanea mollissima* (1 = 100% *C. dentata*)

We estimated hybrid indices, or the proportion of backcross genomes inherited from *C. dentata* v. *C. mollissima*, to quantify the relationship between species ancestry and blight resistance. Hybrid indices varied from 0.99 (99% *C. dentata*) to 0.42 (58% *C. mollissima*) for 865 BC_3_F_2_ descendants of “Clapper,” and from 0.99 to 0.35 for 365 BC_3_F_2_ descendants of “Graves” (Figure 4). There were 24 “Clapper” and 10 “Graves” BC_3_F_2_ trees with hybrid indices less than or equal to 0.55. These trees were inferred to be “pseudo‐F_1_” progeny of BC_3_ mother trees that were pollinated by *C. mollissima* trees on the same property. The average hybrid index of “Clapper” and “Graves” BC_3_F_2_ trees, excluding pseudo‐F_1_s, was 0.89.

### 3.5 Comparison of different selection scenarios

The final step in genomic selection was ranking BC_3_F_2_ selection candidates based on the “Blight Selection Index,” which is the of sum the Parent Condition Index and the Progeny Blight Resistance (see Materials & Methods). We then predicted gains in the “Blight Selection Index” under different selection scenarios. Selection candidates from the “Clapper” and “Graves” populations were planted in 161 and 116 seed orchard plots, respectively. Prior to initial culling, each seed orchard plot contained 150 half‐sib BC_3_F_2_ progeny from an open‐pollinated BC_3_F_1_ mother. Between 1 and 55 trees per plot remained after initial culling (average 5 trees per plot) and were genotyped for genomic selection. We considered three selection scenarios: (1) Select one tree within each seed orchard plot with the largest Blight Selection Index. (2) Select an equal number of trees, but select trees with the largest Blight Selection Index regardless of seed orchard plot. (3) Select the same number of trees, but select a maximum of three trees per plot with the largest Blight Selection Indices. The pseudo‐F_1_ trees were excluded from consideration for selection; however, Blight Selection Indices of the selected trees were compared to that of the pseudo‐F_1_s.

**Figure 5:**
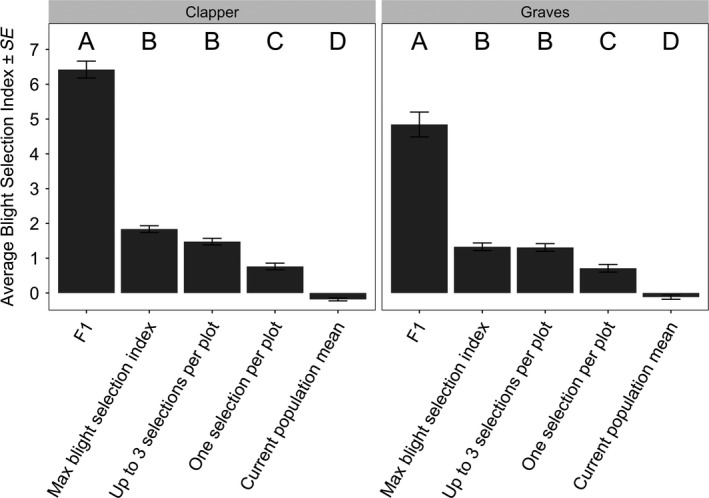
Comparison of average Blight Selection Indices for selected “Clapper” and “Graves” BC_3_F_2_ trees under different selection scenarios. Selection scenarios included making one selection per 150 half‐sibs planted in each seed orchard subplot (one selection per plot); selecting an equal number of trees with the maximum Blight Selection Index (max Blight Selection Index); and making up to three selections per plot (up to three selections per plot). The average Blight Selection Index for the selected BC_3_F_2_ trees was compared to that of the current population and pseudo‐F_1_ trees (i.e., progeny of BC_3_ trees outcrossed to *Castanea mollissima*). Letters above the bars indicate the significance of differences in average Blight Selection Index (Tukey's test, *p* < .05)

For both the “Clapper” and “Graves” populations, all selections scenarios were predicted to increase the mean Blight Selection Index. However, selected trees were, on average, significantly less blight‐resistant than pseudo‐F1s (Figure 5). The average Blight Selection Index of selected BC_3_F_2_ trees was significantly greater when selecting trees with the maximum Blight Selection Index (Scenario 2) or selecting up to three trees per plot (Scenario 3) as compared with selecting one tree per plot (Scenario 1).

The trade‐off when relaxing the constraint of selecting one tree per plot was a reduction of the number of *C. dentata* backcross lineages represented among the selections. For example, the “Clapper” BC_3_F_2_ selection candidates had 41 and 28 *C. dentata* grandparents and great‐grandparents in their maternal line. By selecting 160 trees with the maximum Blight Selection Indices regardless of plot (Scenario 2), selections included descendants from 31 *C. dentata* grandparents and 24 great‐grandparents. By selecting a maximum of three trees per plot, selections included descendants of 33 grandparents and 25 great‐grandparents. We decided to proceed with up to three selections per plot because this scenario resulted in selections with a similar average Blight Selection Indices as Scenario 2 (Figure 5), but retained a larger proportion of the maternal *C. dentata* lineages.

**Figure 6:**
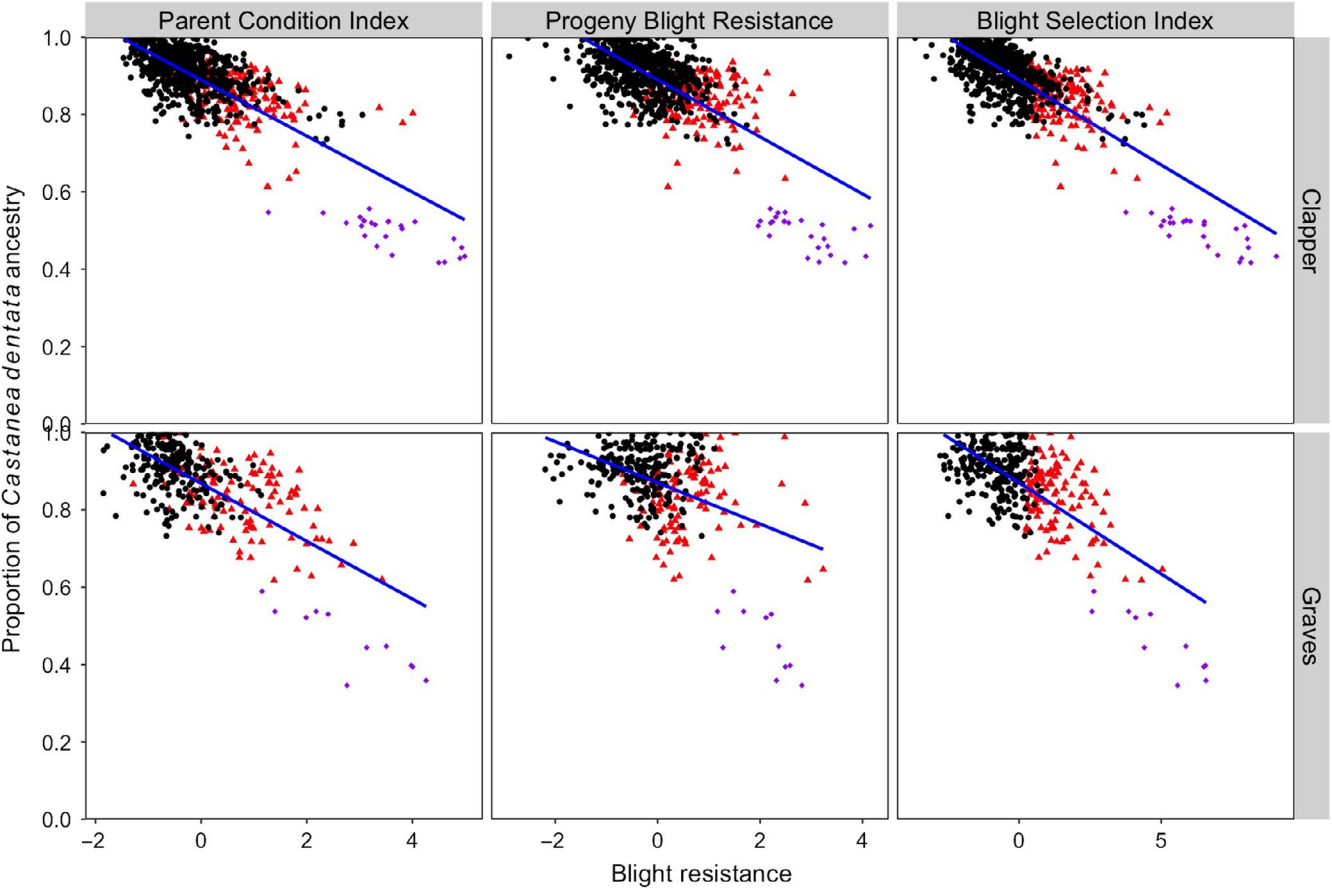
Proportion of Clapper and Graves BC_3_F_2_ genomes inherited from *Castanea dentata* (hybrid index) versus blight resistance. Blight resistance was assessed via the Parent Condition Index (a sum of phenotype probabilities for late‐developing blight trait on BC_3_F_2_ stems), Progeny Blight Resistance (breeding values for average progeny canker severity, reversed in scale), and Blight Selection Index (Parent Condition Index + Progeny Blight Resistance). Red triangles are BC_3_F_2_ selections (up to three selections per 150‐tree subplot), purple diamonds are the pseudo‐F_1_ progeny of BC_3_ trees outcrossed to *Castanea mollissima*, and black dots are inferior trees to cull. Blue lines are the least squares regressions between hybrid index and blight resistance traits

**Figure 7:**
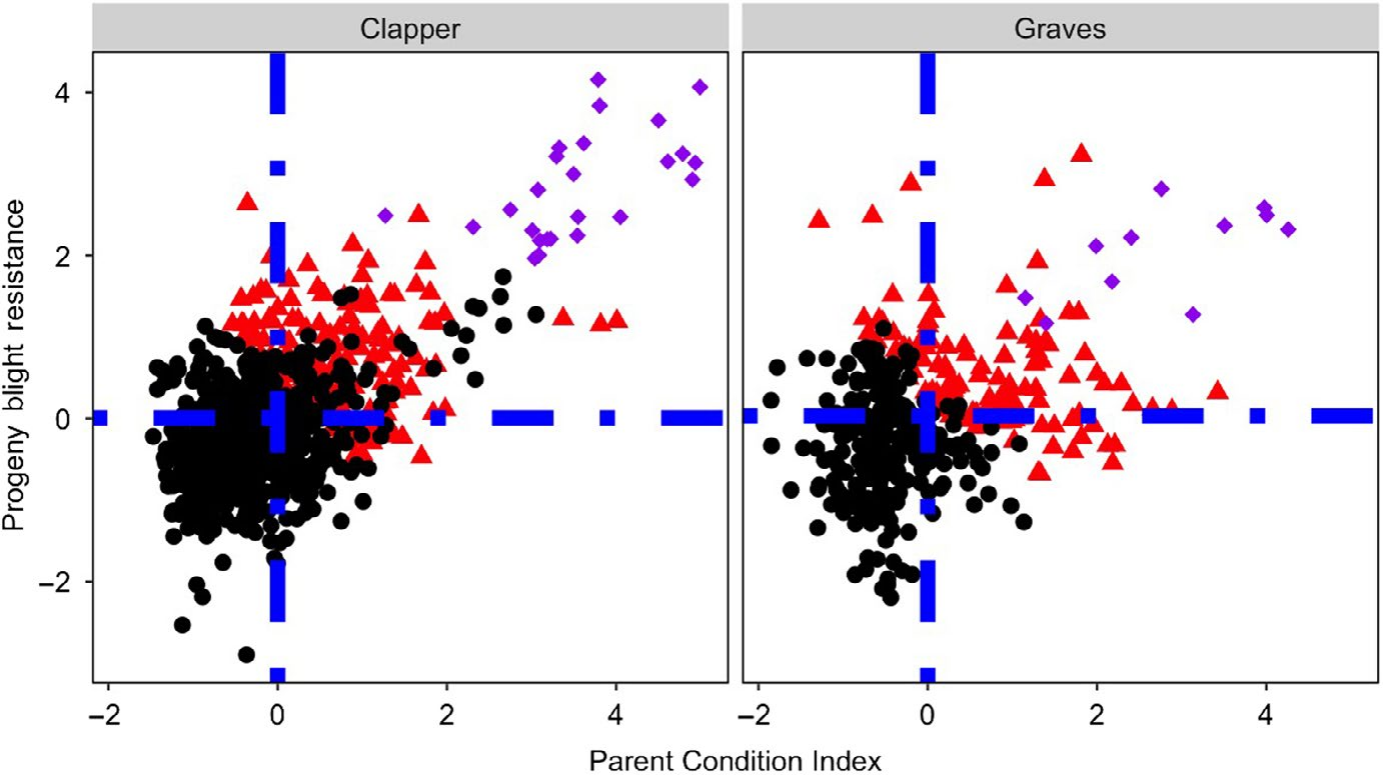
Relationship between Parent Condition Index and Progeny Blight Resistance for “Clapper” and “Graves” populations. Red triangles are BC_3_F_2_ selections with up to three selections per seed orchard plot, purple diamonds are pseudo‐F_1_ progeny of BC_3_ trees outcrossed to *Castanea mollissima*, and black dots are inferior trees to cull. Blue dashed lines are the population means for Parent Condition Index and Progeny Blight Resistance

For both the “Clapper” and “Graves” populations, blight resistance as assessed with the Parent Condition Index, Progeny Blight Resistance, and Blight Selection Index was negatively correlated with the proportion of alleles inherited from *C. dentata* (Figure 6). These negative correlations were observed when genomic prediction models were developed with and without including pseudo‐F_1_s in the training population, suggesting that the pseudo‐F_1_s are not driving this result (not shown). Selected BC_3_F_2_ trees were estimated to have inherited an average (max, min) of 83% (99%, 61%) of their genome from *C. dentata*. Parent Condition Index was positively correlated with Progeny Blight Resistance (“Clapper” *r*_parent‐progeny_ = 0.67, “Graves” *r*_parent‐progeny_ = 0.40; Figure 7). A total of 121 of 161 “Clapper” and 70 of 116 “Graves” selections had above‐average Parent Condition Index and above‐average Progeny Blight Resistance (Figure 7). A representative BC_3_F_2_ selection, a pseudo‐F_1_, and a pure *C. dentata* are pictured in Figure S4.

## 4 DISCUSSION

### 4.1 Outlook for genomic selection for blight resistance in BC_3_F_2_ seed orchards

Our first aim in this study was to optimize genomic selection to increase the speed and accuracy of making final selections for blight resistance in two American chestnut BC_3_F_2_ seed orchards at TACF's Meadowview Research Farms. Previously, approximately 90% of the BC_3_F_2_ in these seed orchards were culled based on blight susceptibility phenotypes after artificial inoculation with *C. parasitica* (Steiner et al., 2017). Our aim with genomic prediction was to accelerate selection of the 1% most blight‐resistant BC_3_F_2_ selection candidates and to circumvent the need to inoculate progeny of all remaining selection candidates to accurately estimate the genetic component of their resistance. We were successful insofar as genomic prediction of late‐developing blight phenotypes of BC_3_F_2_ selection candidates and average canker severity of BC_3_F_3_ progeny were more accurate than prediction from pedigree relationships. Furthermore, all selection scenarios were predicted to increase the average blight resistance relative to the current population mean.

The increased accuracy of genomic prediction may be attributed to more accurate and higher estimates of relatedness between some selection candidates than expected from the pedigree. Genomic prediction accuracy generally increases with increased relatedness between the training and prediction populations (Makowsky et al., 2011; Märtens, Hallin, Warringer, Liti, & Parts, 2016). The genotypic data detected hidden relatedness between BC_3_F_2_ selection candidates, including between the “Clapper” and “Graves” populations. The relatedness between the BC_3_F_2_ selection candidates may be attributed to relatedness among American chestnut founders and full‐sibling relatedness among BC_3_F_2_ trees that were assumed to be half‐sibs in the pedigree analysis. Maximum predictive abilities for average canker severity of BC_3_F_3_ progeny ($r_{y\hat{y}}=0.64$ for “Clapper” and $r_{y\hat{y}}=0.48$ for “Graves”) were similar to or greater than predictive abilities in other forest tree species (e.g., Chen et al., 2018; Isik et al., 2016; Lenz et al., 2019; Resende et al., 2012). The increased predictive ability in this study may be due to increased precision in predicting family means as compared to predicting the phenotypes of individual trees as has been attempted in other studies.

Despite the expanded training population size by combining the “Clapper” and “Graves” populations, the lower or similar predictive ability compared with single‐population analyses suggests that blight resistance segregates at unique loci in the “Clapper” and “Graves” BC_3_F_2_ populations. The “Clapper” and “Graves” BC_1_ sources of resistance may have inherited different portions of their Chinese chestnut grandparents' genomes. Furthermore, the two different Chinese chestnut grandparents may have unique loci for blight resistance. Additional association and QTL analyses of blight resistance are required to test these hypotheses.

We plan to finish selection in the Meadowview seed orchards in the next few years with additional phenotypic selection, progeny testing, and genomic selection. An additional 184 “Clapper” and 216 “Graves” BC_3_F_3_ families will be inoculated in field trials in 2019, 2020, and 2021. Furthermore, genotyping of approximately 1,000 additional “Clapper” and “Graves” BC_3_F_2_ selection candidates is currently ongoing. We anticipate that the accuracy of genomic selection will increase by expanding the training populations as has been predicted from simulation studies (Grattapaglia & Resende, 2011) and observed for other species and traits (Asoro, Newell, Beavis, Scott, & Jannink, 2011; Chen et al., 2018; Zhang et al., 2017).

### 4.2 Evaluating the genetic architecture of blight resistance

Two observations suggest that chestnut blight resistance is inherited as a polygenic trait. First, we observed a trade‐off between blight resistance and the proportion of BC_3_F_2_ trees' genomes inherited from *C. dentata*. Second, HBLUP, which assumes an infinitesimal model of inheritance, was just as accurate at predicting progeny canker severity as Bayes C, which includes only the markers with largest effects in the prediction model. Previous QTL mapping studies of blight resistance were conducted in a small *C. dentata* × *C. mollissima* F_2_ family (<100 full‐sib progeny; Kubisiak et al., 1997, 2013); therefore, it is likely that the effects of individual loci were inflated and these studies were underpowered to comprehensively detect all loci associated with blight resistance (Beavis, 1994; Slate, 2013). Quantitative resistance to *C. parasitica* is plausible considering that polygenic inheritance has been detected for other plant species' resistance to necrotrophic pathogens (see review by Corwin & Kliebenstein, 2017). Regardless of the number of loci underlying blight resistance, the low heritabilities (*h* ^2^ < 0.25) of blight resistance phenotypes of suggest that some alleles for blight resistance have been lost in some backcross generations and lines as a result of low‐accuracy phenotypic selection.

### 4.3 Revised projections of average blight resistance after selection at BC_3_F_2_

Steiner et al. (2017) predicted that final selection in BC_3_F_2_ seed orchards would result in a BC_3_F_3_ population with an average blight resistance similar to *C. mollissima* × *C. dentata* F_1_ hybrids. We observed that average blight resistance of BC_3_F_2_ selections that inherited approximately 90% of their genome from *C. dentata* was less than that of pseudo‐F_1_ trees, which inherited approximately 50% of their genome from *C. dentata*. Previous studies have found that BC_3_F_3_ progeny from partially selected seed orchards have improved blight resistance relative to *C. dentata* in orchard and greenhouse trials (Steiner et al., 2017; Westbrook & Jarrett, 2018). Therefore, we predict that the average blight resistance of BC_3_F_3_ progeny from fully selected BC_3_F_2_ seed orchards will be between that of F_1_ hybrids and *C. dentata*.

### 4.4 Where does breeding for American chestnut restoration go from here?

#### 4.4.1 Restoration trials

It is not known what combination of blight resistance and *C. dentata* inheritance will be sufficient for American chestnut restoration. The American Chestnut Foundation has planted field trials composed of BC_3_F_3_ progeny from Meadowview seed orchards at over 35 sites across the eastern United States (Figure 1). Many of these trials are between 5 and 10 years old: too young to reliably assess for blight resistance following natural infection by *C. parasitica*. Encouragingly, in the oldest field trials, blight incidence and severity on 8‐year‐old BC_3_F_3_ trees were lower than on pure American chestnut and similar to Chinese chestnut (Clark, Schlarbaum, Saxton, & Baird, 2019). Once selection is complete in seed orchards, TACF intends to plant additional restoration trials composed of the most blight‐tolerant BC_3_F_3_ families planted on sites most suitable for growing American chestnut. The influence of environmental factors such as competition, climate, and soil on blight resistance will be estimated via replication of BC_3_F_3_ families across sites and varying silvicultural treatments within sites (TACF, 2012).

#### 4.4.2 Breeding strategies to improve blight resistance

Blight resistance may be improved with additional generations of intercrossing and recurrent selection (Westbrook, 2018). Once resistance is sufficient for backcross trees to compete and reproduce in forests, natural selection may continue to improve resistance and competitive ability. Furthermore, the American Chestnut Foundation is currently generating and selecting *C. dentata* backcross progeny from additional *C. mollissima* sources of blight resistance (Steiner et al., 2017; Westbrook, 2018). Based on the finding of a trade‐off between blight resistance and *C. dentata* inheritance, we will advance these additional sources only to the BC_1_ or BC_2_ generations rather than BC_3_ before intercrossing the selections. Backcross trees will be selected for blight resistance not only with phenotypic selection, but also by inoculating progeny derived from controlled pollinations of these trees to ensure that selection is accurate.

While BC_1_ and BC_2_ selections are expected to be more blight‐tolerant than selections from later backcross generations, the earlier backcross selections are expected to inherit other traits from *C. mollissima* that may be undesirable for forest restoration. Compared with American chestnut, Chinese chestnuts growing in North America generally have lower height growth (Diller & Clapper, 1969; Schlarbaum et al., 1998; Thomas‐Van Gundy, Bard, Kochenderfer, & Berrang, 2016), greater stem branching (Clark, Sclarbaum, Saxton, & Hebard, 2012), lower maximum photosynthetic rates (Knapp, Wang, Clark, Pile, & Sclarbaum, 2014), lower cold tolerance (Gurney, Schaberg, Hawley, & Shane, 2011; Saielli, Schaberg, Hawley, Halman, & Gurney, 2012), and differential colonization of roots by mycorrhizae and other fungi (Reazin, Baird, Clark, & Jumponnen, 2019). After selecting for blight resistance, we will perform additional selection for timber‐type form and overall proportion of backcross trees' genomes inherited from *C. dentata*, which will necessitate screening large populations segregating for these traits.

#### 4.4.3 Incorporating transgenic blight resistance

Lower than expected blight resistance within BC_3_F_3_ populations highlights potential advantages of using transgenic American chestnut trees for restoration. Transgenic *C. dentata* founder lines that constitutively overexpress an oxalate oxidase (OxO) gene from wheat have high levels of blight resistance in seedling trials (Newhouse et al., 2014; Powell, Newhouse, & Coffey, 2019). Transgenic varieties have the *C. dentata* genetic background except one gene that confers blight tolerance. When transgenic *C. dentata* are crossed with wild‐type trees, 50% of their progeny are expected to inherit the resistance gene, which can be detected inexpensively with an enzymatic assay or with PCR (Zhang et al., 2013). Federal regulatory review in the United States is ongoing to release transgenic American chestnut founder trees for breeding and restoration trials outside of a few confined, permitted field trials. If federal regulatory approval is granted, TACF plans to outcross transgenic founder clone(s) to wild‐type trees over five generations to increase the effective population size to >500 and to maximize genome inheritance from wild‐type trees with marker‐assisted introgression (Westbrook, Holliday, Newhouse, Powell, 2019a). Transgenic trees may also be crossed with backcross trees to potentially enhance blight resistance. Public acceptance of transgenic American chestnut trees for restoration is mixed (Delborne et al., 2018), and the long‐term blight resistance of transgenic trees in forest conditions is not currently known. Therefore, it is prudent to continue traditional breeding approaches that are informed by genomics separately from breeding with transgenic trees.

## 5 CONCLUSIONS AND FUTURE DIRECTIONS

In developing genomic prediction models and estimating hybrid indices for BC_3_F_2_ American chestnuts, we discovered a trade‐off between blight resistance and proportion of the genome inherited from *C. dentata*. Results suggest that genetic architecture underlying the inheritance of blight resistance is more complex than previously assumed. A chromosome‐scale genome assembly for *C. dentata* is forthcoming, which will be combined with genotyping of thousands of backcross individuals to enable mapping the inheritance of *C. mollissima* haplotypes and discovery of genomic regions associated with variation in blight resistance.

## CONFLICT OF INTEREST

None declared.

## Data Availability

Demultiplexed and quality‐trimmed sequence reads per sample have been uploaded to the NCBI Sequence Read Archive (SRA) under BioProject accessions https://www.ncbi.nlm.nih.gov/bioproject/PRJNA507748 and https://www.ncbi.nlm.nih.gov/bioproject/PRJNA507747. The blight phenotypes, pedigree files, and a VCF file containing the nonimputed and imputed SNPs can be accessed on Dryad (Westbrook, 2019b, https://doi.org/10.5061/dryad.8w9ghx3gn). Contact Jeremy Schmutz (jschmutz@hudsonapha.org) for access to the latest assembly of the *C. dentata* genome sequence under the Ft. Lauderdale Agreement. Once the annotation is finalized, the genome will be publicly available at the Phytozome, comparative plant genomics portal.
