# Supplementary figures and images for: Optimizing genomic selection for blight resistance in American chestnut backcross populations: A trade‐off with American chestnut ancestry implies resistance is polygenic
Source: Evol Appl. 2019 Dec 29;13(1):31–47. doi: 10.1111/eva.12886 (PMC6935594; doi:10.1111/eva.12886)

## Slide 1
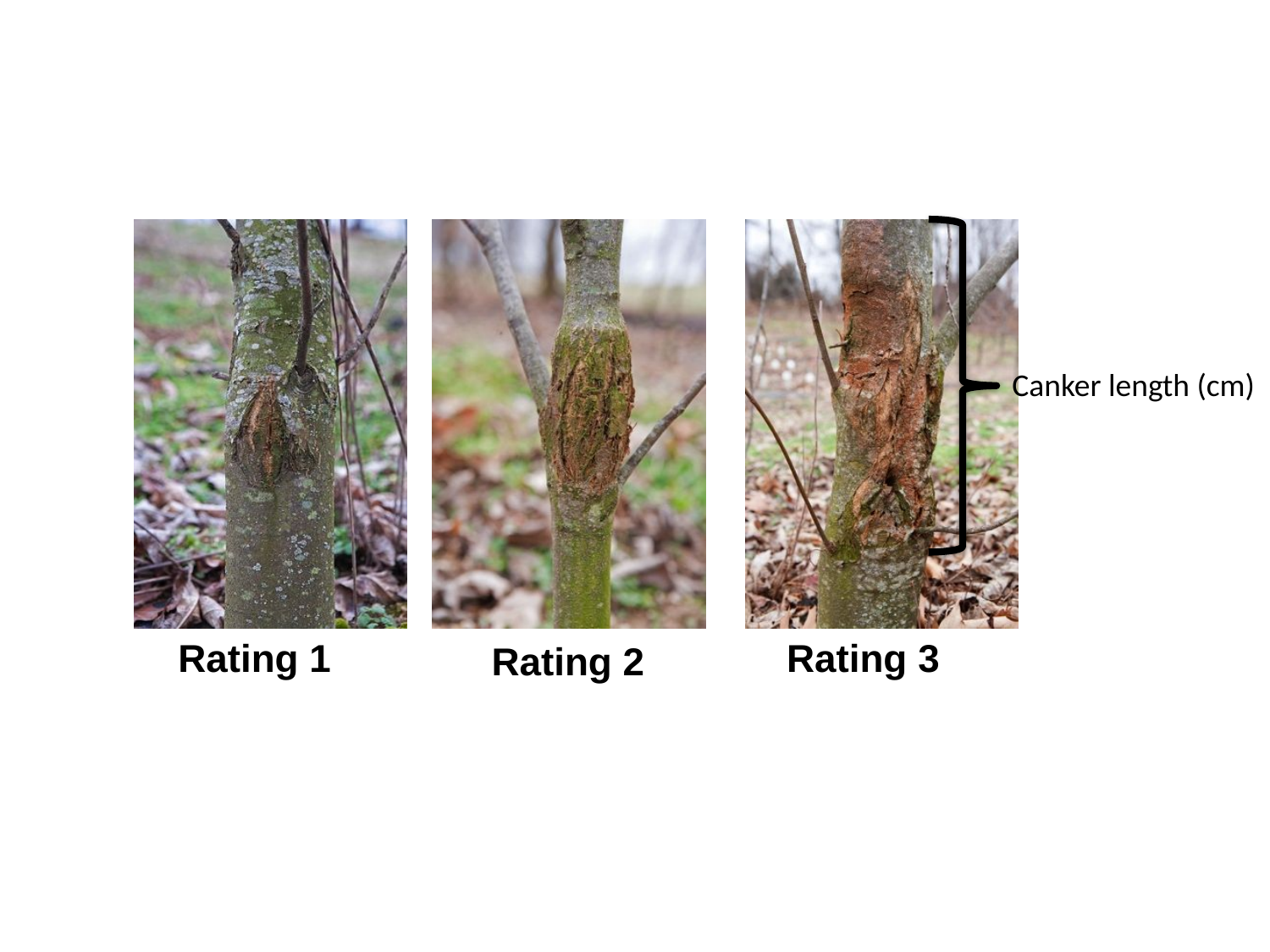

Canker length (cm)
Rating 1
Rating 3
Rating 2

Supplement: Supplementary file 1 [file EVA-13-31-s001.zip › eva12886-sup-0001-FigS1.pptx]

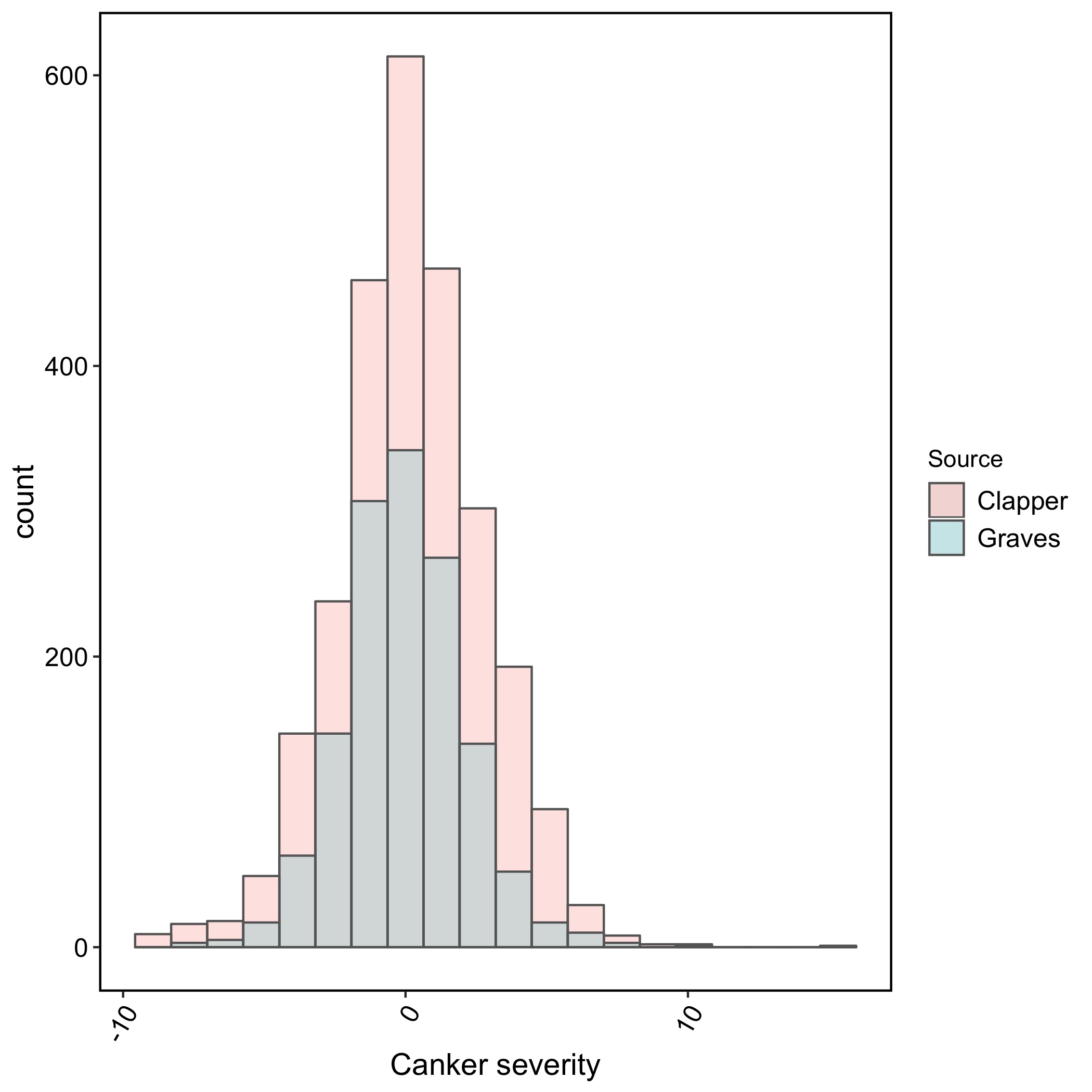

Supplement: Supplementary file 1 [file EVA-13-31-s001.zip › eva12886-sup-0002-FigS2.jpeg]

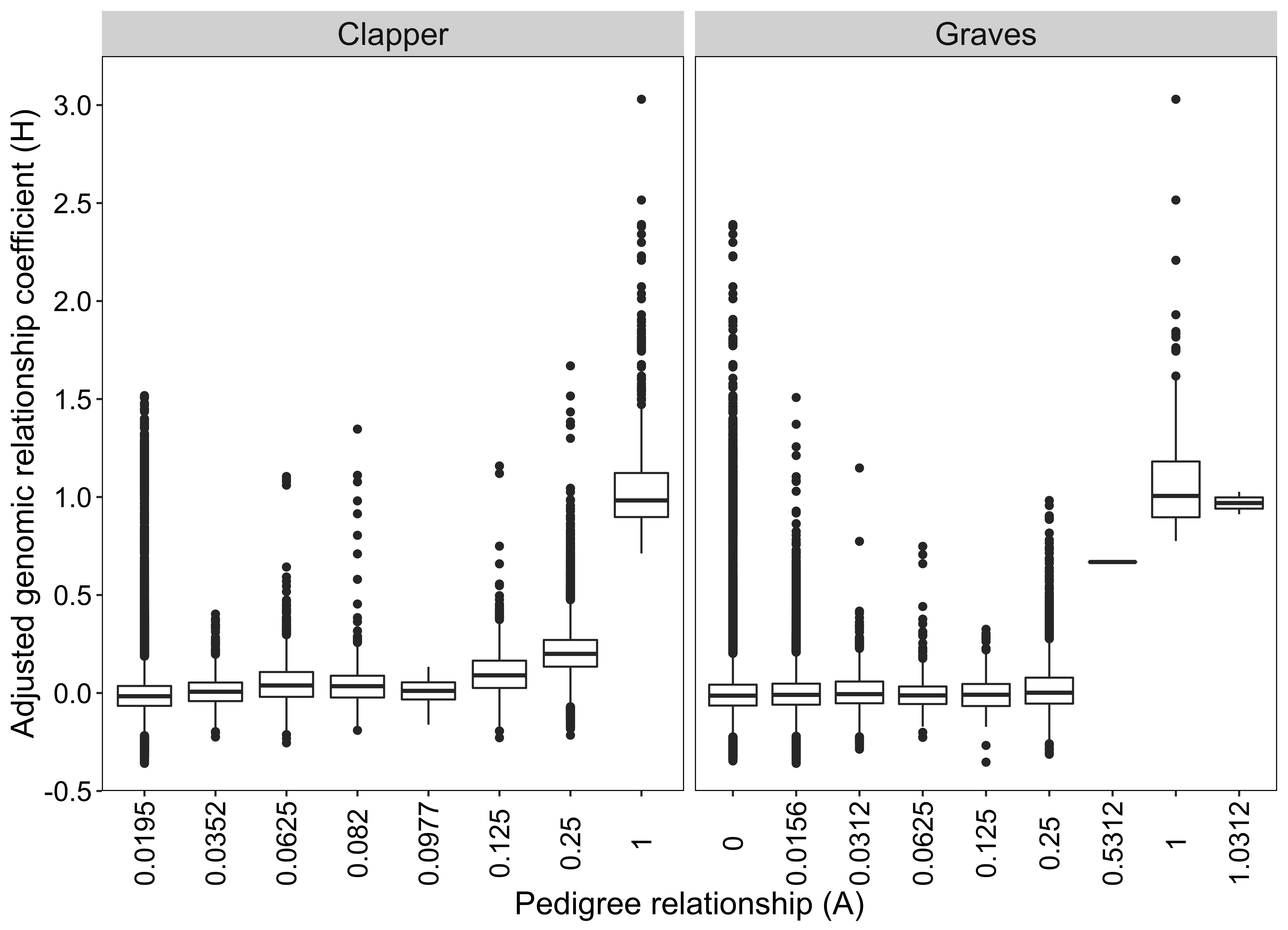

Supplement: Supplementary file 1 [file EVA-13-31-s001.zip › eva12886-sup-0003-FigS3.jpeg]

## Slide 1
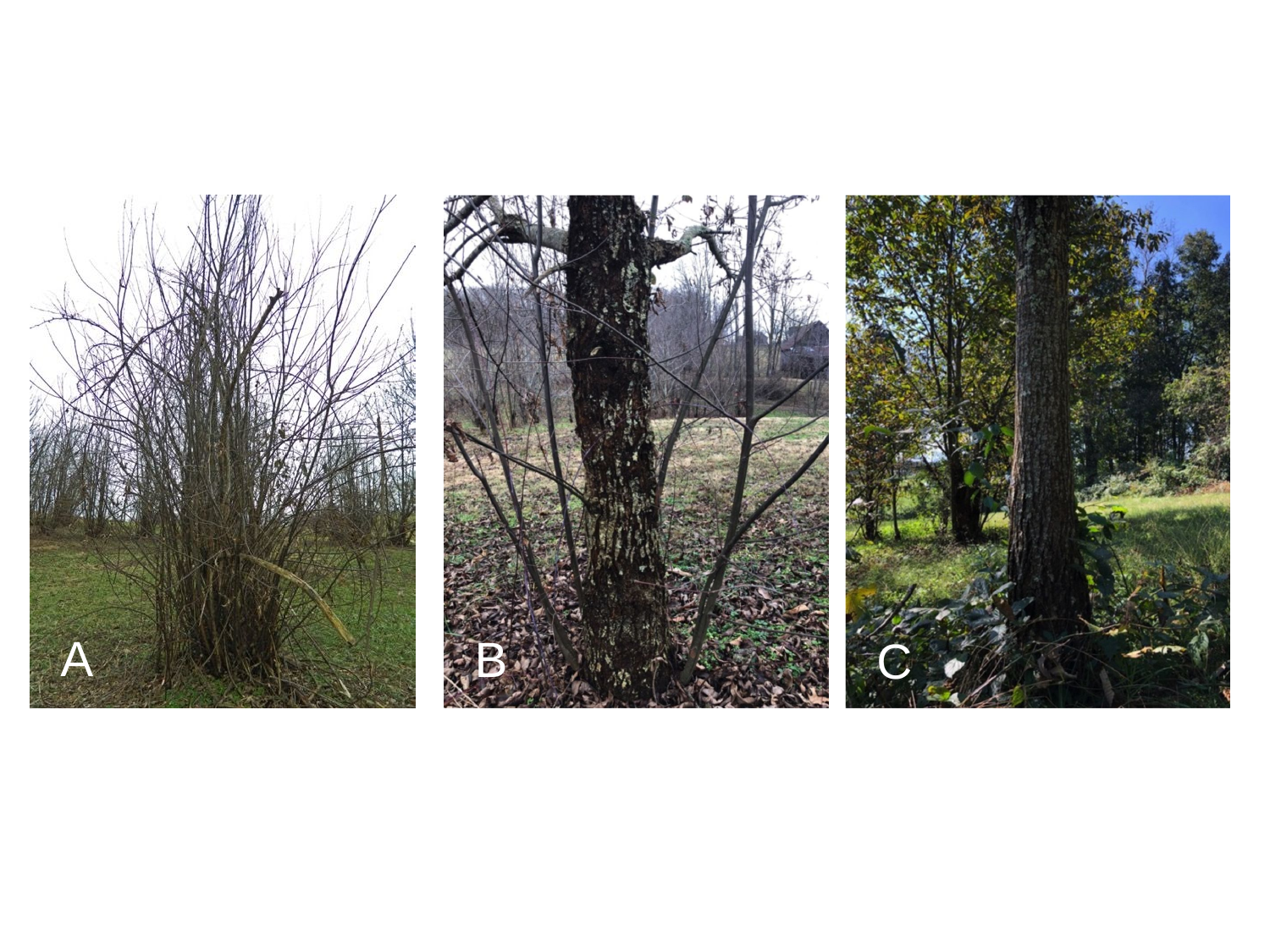

A
B
C

Supplement: Supplementary file 1 [file EVA-13-31-s001.zip › eva12886-sup-0004-FigS4.pptx]
